# A Retrospective Comparison of Medication Recommendations Between a Cardiologist and ChatGPT-4 for Hypertension Patients in a Rural Clinic

**DOI:** 10.7759/cureus.55789

**Published:** 2024-03-08

**Authors:** Ghaith Al Tibi, Melvin Alexander, Samuel Miller, Nicolas Chronos

**Affiliations:** 1 College of Medicine, Albert Einstein College of Medicine, Bronx, USA; 2 College of Medicine, Rush University Medical Center, Chicago, USA; 3 Cardiology, Lake Country Medical Group, Eatonton, USA

**Keywords:** retrospective comparative study, chatgpt in healthcare, lab interpretation, medication management, large language model, rural setting, chatgpt-4

## Abstract

Background

With ChatGPT demonstrating impressive abilities in solving clinical vignettes and medical questions, there is still a lack of studies assessing ChatGPT using real patient data. With real-world cases offering added complexity, ChatGPT’s utility in treatment using such data must be tested to better assess its accuracy and dependability. In this study, we compared a rural cardiologist’s medication recommendations to that of GPT-4 for patients with lab review appointments.

Methodology

We reviewed the lab review appointments of 40 hypertension patients, noting their age, sex, medical conditions, medications and dosage, and current and past lab values. The cardiologist’s medication recommendations (decreasing dose, increasing dose, stopping, or adding medications) from the most recent lab visit, if any, were recorded for each patient. Data collected from each patient was inputted into GPT-4 using a set prompt and the resulting medication recommendations from the model were recorded.

Results

Out of the 40 patients, 95% had conflicting overall recommendations between the physician and GPT-4, with only 10.2% of the specific medication recommendations matching between the two. Cohen’s kappa coefficient was -0.0127, indicating no agreement between the cardiologist and GPT-4 for providing medication changes overall for a patient. Possible reasons for this discrepancy can be differing optimal lab value ranges, lack of holistic analysis by GPT-4, and a need for providing further supplementary information to the model.

Conclusions

The study findings showed a significant difference between the cardiologist’s medication recommendations and that of ChatGPT-4. Future research should continue to test GPT-4 in clinical settings to validate its abilities in the real world where more intricacies and challenges exist.

## Introduction

Ever since ChatGPT was launched in November of 2022, there has been a heightened interest in the use of large language models (LLMs). Various industries have tried to incorporate ChatGPT to enhance their line of work. One such industry is healthcare, where numerous projects have been conducted to assess ChatGPT’s abilities in tasks such as medical writing, triage, and research [1-3]. This artificial intelligence (AI) model has also been tested using clinical vignettes and questions, showing impressive accuracy in its answers [4,5]. Moreover, one study found that ChatGPT was able to pass the United States Medical Licensing Examination Step 1 test [6]. With such proven abilities in answering medical questions and cases, the question remains whether ChatGPT could be of utility for clinicians of various specialties when it comes to diagnosis and treatment. Such AI models could offer instant medical information to both patients and providers in rural settings with limited access to physician specialists and medical resources.

Currently, there is a lack of studies assessing ChatGPT using real patient data. With real-world cases offering added complexity, ChatGPT’s utility in treatment using such data must be tested to better assess its accuracy and dependability. Our study aimed to compare a rural physician specialist’s evaluation of patient data, as done during lab review appointments, with that of ChatGPT’s evaluation. Specifically, we used medication recommendations as a parameter for comparing the evaluations of the physician and ChatGPT. With hypertension being a prominent condition in rural areas and one with several comorbidities [7,8], we chose patients with hypertension as our population for this study to acquire complex cases that are relevant to rural settings. We used ChatGPT’s latest model, ChatGPT-4 (GPT-4), to conduct the study as it has proven to be much more accurate in solving clinical questions than previous models [9]. If GPT-4 can produce medically accurate recommendations, this will have a profound effect on the future of patient treatment. Additionally, it can be especially important in rural settings where the effects of physician shortage can be ameliorated through the use of LLMs for medical consults.

## Materials and methods

Patient selection

We reviewed the charts of 200 patients with hypertension at Lake Country Medical Group in rural Georgia. Inclusion into the study required patients to have at least two serum lab review appointments with the clinic’s cardiologist. At least two lab appointments were required to have “current” and “past” lab values for GPT-4 to reference when recommending medication changes, similar to how a physician looks at past values in a lab review. Of the 200 patients, 40 were selected for the study based on the inclusion criteria.

Data collection

For each patient, the following data was collected: age, sex, medical conditions, medications and dosage, and current and past labs with their respective dates. Labs included the following markers: high-sensitivity C-reactive protein (hsCRP), microalbumin/creatinine, alkaline phosphatase, aspartate transaminase (AST), alanine transaminase (ALT), fibrosis-4 (FIB-4), magnesium, uric acid, thyroid-stimulating hormone (TSH), thyroxine (T4), triiodothyronine (T3), ferritin, iron, white blood cell (WBC), red blood cell (RBC), hemoglobin (Hgb), and severe acute respiratory syndrome coronavirus 2 (SARS-CoV-2) antibody. The recorded data is consistent with the information usually found on a lab review chart and includes the essential information that a physician inspects when reviewing serum lab tests. The cardiologist’s medication recommendations (stopping, decreasing dose, increasing dose, or adding medications) from the most recent lab visit, if any, were recorded for each patient.

Input of patient data into GPT-4

The following prompt was introduced into GPT-4 for each patient: “Provide medication changes if needed for this patient based on most recent lab values, taking into consideration patient’s age, sex, medical conditions, medications, and past lab values. Organize recommendations in the following manner: Medications that may need to be stopped, medications that may need to be lowered in dose, medications that may need to be increased in dose, and medications that may need to be added. For each category, if no change needs to be implemented, write N/A.” The prompt was followed by patient data inputted in the following manner: “Patient is a (age) year old (sex) with (medical conditions) taking the following medications: (medications and dosage). Labs (date): inflammatory marker - hsCRP (value), microalbumin/creatinine (value), cholesterol (value) - HDL (value), LDL (value), TG (value), sugars - A1c (value), vitamin D (value), omega-3 (value), BUN (value), creatinine (value), alk phos (value), AST (value), ALT (value), FIB-4 (value), magnesium (value), uric acid (value), thyroid - TSH (value), T4 (value), T3 (value), ferritin (value), iron (value), hematology - WBC (value), RBC (value), Hgb (value), SARS-CoV-2 antibody (value).” The output response from GPT-4 was recorded.

Analysis of recommendations

Medication recommendations by both the cardiologist and GPT-4 were categorized into four groups: medications to stop, medications to lower in dose, medications to increase in dose, and medications to add. Recommendations were compared between the physician and GPT-4 for each of these categories as well as overall. Two-tailed paired t-tests were used to determine if significant differences exist between the various comparisons. Concordance analysis was done using agreement/disagreement rates to provide medication recommendations for each patient and evaluated using Cohen’s kappa.

## Results

Medication recommendations for patients were successfully collected from both the cardiologist and GPT-4. Results were compared and a breakdown can be seen in Table 1. Of the 40 patients, 95% had conflicting overall recommendations between the physician and GPT-4. The remaining 5% were cases in which recommendations were not provided by either the physician or GPT-4. Specific medications involved in the recommendations by either source are provided in Table 2. Overall, when agreement and disagreement rates were compared for providing medication changes overall for a patient, Cohen’s kappa coefficient was -0.0127, with values <0 indicating no agreement.

**Table 1:** Medication recommendations by GTP-4 vs. cardiologist. *: Results were significantly different (p < 0.05) between the physician and GPT-4 in that category.

	Stop	Decrease	Increase	Add	Total
Physician	0*	0*	7	42*	49*
GPT-4	16*	16*	8	62*	102*
Percent match	0%	0%	6.70%	12.50%	10.20%

**Table 2:** Types of medications involved in recommendations by GTP-4 vs. cardiologist.

	Types of medications
Physician	Antidiabetics, antilipemics, beta-blockers, omega-3, vitamin D, vaccinations
GPT-4	Alpha-blockers, angiotensin receptor blockers, antianemics, antibiotics, anticholinergics, anticoagulants, anticonvulsant, antidepressants, antidiabetics, antifungals, antihistamines, antihypertensives, antilipemics, antiplatelets, anxiolytics, beta-blockers, bronchodilator, calcium, folic acid, hormones, hypnotics, iron, nitrate, non-steroidal anti-inflammatory drugs, omega-3, phosphodiesterase inhibitors, potassium, proton pump inhibitors, vaccinations, vitamin B12, vitamin D, xanthine oxidase inhibitors

## Discussion

The results from the study indicate clear differences between the cardiologist’s medication recommendations and those of GTP-4 in multiple aspects. Overall, GPT-4 made a greater number of medication changes compared to the physician with more than double the recommendations. GTP-4 had significantly more recommendations involving stopping, adding, and decreasing the dosage of medications, while those involving increasing the dosage of medications were not significantly different from the physician. With Cohen’s kappa coefficient being less than 0, GTP-4 and the cardiologist were not in agreement when deciding whether to make a medication change or not for patients. Primarily, these results are indicative of GTP-4 having a greater propensity to implement medication changes for patients in comparison to a cardiologist.

Not only did GTP-4 and the cardiologist differ in the quantity and propensity of their recommendations they also differed in the kinds of medications involved in these recommendations. The only times both sources agreed on how to manage a patient was when no medication recommendations were given at all. However, every time medication recommendations were given for a patient, at least one of the medications involved was different between GTP-4 and the physician. With only 10.2% of the individual medication recommendations matching, there was a low consensus on how to approach these patients’ lab values. Moreover, when examining the specific classes of medications, GTP-4’s recommendations offered a far greater diversity in medications spanning over 30 types, while the cardiologist alternated between six main classes of medications.

Possible reasons for differences

In trying to understand why these differences exist between the two, we can look at a specific patient case. A 70-year-old male patient with type II diabetes, hyperlipidemia, and hypertension had blood work done most prevalently showing high-density lipoprotein at 38 mg/dL (previously 49 mg/dL), low-density lipoprotein at 74 mg/dL (previously 59 mg/dL), triglycerides at 168 mg/dL (previously 89 mg/dL), HbA1C at 8.8% (previously 6.7%), vitamin D at 48.5 ng/mL (previously 46.3 ng/mL), and omega-3 at 4.5% (previously 3.9%). For this patient, both the cardiologist and GPT-4 recommended the addition of antidiabetic medication. However, the physician also recommended the addition of vitamin D and omega-3 supplements. Optimal vitamin D levels, similar to other markers, have been a topic of discussion and continuous debates occur to determine its recommended values [10]. While some may find the patients’ values of vitamin D and omega-3 to be sufficient, the physician prefers values of vitamin D to be above 50 ng/mL and omega-3 above 5.5%. On the other hand, GPT-4 recommended starting an antilipemic medication. While his lipid panel did worsen, so did his HbA1C. His high sugar levels may be playing a role in the worsening of his lipid values and thus might improve when sugar levels are under control, which has been well understood to occur in patients with diabetes mellitus [11]. Moreover, when asked about his diet, the patient indicated to the physician about not having an optimal one. As such, he was given a chance to improve his lipid levels through diet as well as by controlling his blood sugar. GTP-4 was not provided information on the patient’s diet and might have assumed it was well-maintained. Finally, GTP-4 also recommended stopping Zyrtec which the patient had been using for his allergies. Seasonal allergies were not included as part of the patient’s medical history in the patient’s chart and thus were recommended to be removed as GPT-4 did not see Zyrtec as serving a purpose. The cardiologist, knowing the patient, knew that the over-the-counter medication was being used for this purpose without having seasonal allergies listed between the patient’s medical conditions.

This patient case can be highly informative in describing possible reasons for discrepancies between the recommendations of the cardiologist and GPT-4. One reason, as seen with vitamin D and omega-3, might be a difference in desired optimal lab values. Another, as in the case of the lipid levels, might be a lack of holistic analysis where a value was taken as high without interpreting that value as it relates to other components of the lab test. Additionally, a lack of supplementary information such as the diet of the patient prevented GPT-4 from making the appropriate recommendation. These ideas have generally been replicated in previous work which concluded that GPT-4 was able to analyze lab results on a test-by-test basis, but unable to appraise values in the context of the patient’s full medical picture [12]. Finally, the cardiologist’s familiarity with the patient, as with Zyrtec, provided an added advantage to making optical medical suggestions. This coincides with data on consumer receptivity to AI in medicine. It has been found that patients are reluctant to utilize healthcare provided by AI in real and hypothetical choices and feel that AI providers are less able than human providers to account for their unique characteristics and circumstances [13]. Future research should continue to test GPT-4 in clinical settings. While this LLM has proven to be impressive in answering medical test questions and medical case conferences, its capabilities need to be further validated in the real world where more complexity and challenges exist.

Study limitations and future directions

While the strengths of this study lie in its use of real clinical data to evaluate ChatGPT’s performance and its direct comparison to a physician’s clinical decision-making, it is important to acknowledge the limitations of the study. This study was conducted at a single center with one physician. It also assumes that the physician’s recommendations are inherently correct and does not account for physician variability in clinical practice. In addition, the sample size was only 40 patients selected from the same clinic. With ChatGPT only being provided input from the cardiologist’s notes and labs, its recommendations were restrained by this limited information. Overall, these limitations affect the generalizability and accuracy of the results. Future research should aim to overcome these limitations by analyzing recommendations from multiple health centers and providers and increasing the sample size. It might also be beneficial to provide ChatGPT with the real-time conversation between the physician and the patient to have as much information available to it as the physician when making their recommendations.

## Conclusions

This study shows a significant difference between the cardiologist’s medication recommendations and that of ChatGPT-4. The LLM also had a greater propensity to recommend changes involving a wider variety of medication classes. In patient cases where medication recommendations were made by both, GPT-4’s changes were mostly inconsistent with the cardiologist’s recommendations. Possible reasons for this might include variations in what is considered optimal lab value levels between this physician and the model. In addition, GPT-4’s lack of access to supplementary information, unfamiliarity with the patients, and a deficient systemic analysis compared with the cardiologist may be leading to the observed differences.
